# Comparison of Propofol and Sevoflurane as Anesthesia Maintenance Agents in Relation to Postoperative Pain, Nausea and Vomiting During Endoscopic Retrograde Cholangio-Pancreatography: A Prospective, Single-Center, Randomized, Single-Blind Pilot Study

**DOI:** 10.7759/cureus.81627

**Published:** 2025-04-02

**Authors:** Mehdi Belhassane, Leo Jaubert

**Affiliations:** 1 Anesthesiology and Reanimation, Université Libre de Bruxelles, Brussels, BEL; 2 Anesthetics, Hôpital Universitaire de Bruxelles (HUB) - Erasme Hospital, Brussels, BEL

**Keywords:** endoscopic retrograde cholangiopancreatography (ercp), postoperative nausea and vomiting, postoperative pain, sevoflurane vs propofol, tci

## Abstract

Introduction

Halogen gas anesthesia is considered to be more conducive to postoperative nausea and vomiting (PONV) than total intravenous anesthesia. This difference has been demonstrated in surgery, but there have been no studies on postoperative pain (POP), nausea and vomiting based on anesthesia in the specific environment of endoscopy, to the best of our knowledge. Our aim was therefore to carry out a preliminary study to investigate a trend in POP, nausea and vomiting during endoscopic retrograde cholangio-pancreatography (ERCP), depending on the type of the maintenance anesthetic agent used.

Methods

This single-center, prospective, randomized, single-blind pilot study included 42 adult patients (American Society of Anesthesiologists classification score, ASA ≤ 3) benefiting from ERCP. They were randomized into two groups depending on the type of maintenance anesthetic used: the “sevoflurane” group and the “propofol” group (target-controlled infusion, Schnider model). Moreover, in both groups, all patients received balanced anesthesia based on alfentanil and rocuronium. The principal aim of our study was to investigate pain, nausea and vomiting during the first 48 hours postoperatively based on the anesthesia maintenance agent chosen. During the first 48 hours, PONV episodes were counted, pain was measured using the visual analogue scale and all analgesics and antiemetics administered were recorded.

Results

The two groups studied showed no statistically significant difference. The incidence of PONV was 36.4% in the sevoflurane group and 30% in the propofol group. Statistical analysis showed no significant difference in the occurrence of PONV or in the number of emetic episodes at 48 h postoperatively. There was no significant difference between the two groups in terms of the analgesics used to assess postoperative pain. Finally, there were no differences in the types of analgesics used postoperatively.

Conclusion

There appeared to be no difference in terms of postoperative pain, nausea and vomiting after ERCP in adults, based on the choice of maintenance anesthetic agent (sevoflurane versus propofol). However, the results of this pilot study should be confirmed in a larger sample.

## Introduction

The practice of anesthesia dates back to ancient times, and it was not until the mid-19th century that it entered its modern era, separating the surgical act from pain. Since then, there has been a succession of anaesthetics, each with its own set of more or less severe postoperative complications. Over time, however, anaesthetic products have become increasingly safe, despite the persistence of certain adverse effects [1,2].

Indeed, postoperative nausea and vomiting (PONV) and postoperative pain (POP), which can contribute to the increased incidence of PONV, are frequent and undesirable complications in patients undergoing procedures requiring general anesthesia. PONV affects 20% to 30% of patients, with the rates reaching as high as 80% when risk factors are present [2-5]. PONV can be severe in 0.1% of cases, and it may be complicated by electrolyte disorders, esophageal injury, inhalation pneumonitis, or wound dehiscence, which can lead to additional costs and prolonged hospitalization [6]. Inhaled anesthesia, which combines induction with propofol and maintenance with sevoflurane, has been shown to increase the incidence of PONV, compared to total intravenous anesthesia (TIVA) with propofol alone [4,6,7]. This difference is consistent with study findings on certain open and laparoscopic surgeries [6,8,9,10]. However, there are very few studies comparing these two types of anesthesia in relation to PONV, and to our knowledge, there have been no studies addressing the differences in the onset of PONV and POP depending on the type of anesthesia chosen in the specific setting of interventional gastroenterology. Interventional endoscopy encompasses a number of procedures. One of them is endoscopic retrograde cholangio-pancreatography (ERCP), a diagnostic and therapeutic procedure that enables minimally invasive management of pancreatic and biliary pathologies, with a complication rate of around 10%, which increases when risk factors are present [11,12]. ERCP utilizes the natural digestive tract, considerably limiting tissue damage and postoperative opioid use, and also requires shorter anesthesia times, which may contribute to a reduced incidence of PONV [5].

The main objective of this study was therefore to determine, in the specific setting of interventional endoscopy, whether there is a difference between sevoflurane and propofol as maintenance anesthetic agents in relation to the occurrence of postoperative pain, nausea, and vomiting during uncomplicated ERCP.

## Materials and methods

Study design and sampling

This prospective, single-center, randomized, single-blind pilot study was approved by the Ethics Committee of our hospital (reference P2018/610/CCB B406201838158), and was carried out in the Anesthesia-Resuscitation Department of Hôpital Erasme between January and May 2020. Patients were assigned to two groups using randomization: “sevoflurane” group where induction was done with propofol and maintenance with sevoflurane 2%, and “propofol” group, where induction and maintenance were carried out using propofol via a target-controlled infusion (TCI) device. Patients were unaware of the anesthesia they were about to receive, as the operators were informed a few minutes before induction. Inclusion and exclusion criteria are listed in Table 1 (see, also, the Appendices).

**Table 1:** Criteria for inclusion of patients in the study ASA, American Society of Anesthesiologists; GERD, gastroesophageal reflux disease; PUD, peptic ulcer disease; ERCP, endoscopic retrograde cholangio-pancreatography

Inclusion criteria	Exclusion criteria
Age >18 years	Contraindication to the anaesthetics used or mental and cognitive pathologies
ASA ≤3, eligible for ERCP	Pregnancy or emetogenic pathology except GERD and PUD, patients undergoing chemotherapy

Description of the final cohort

**Figure 1:**
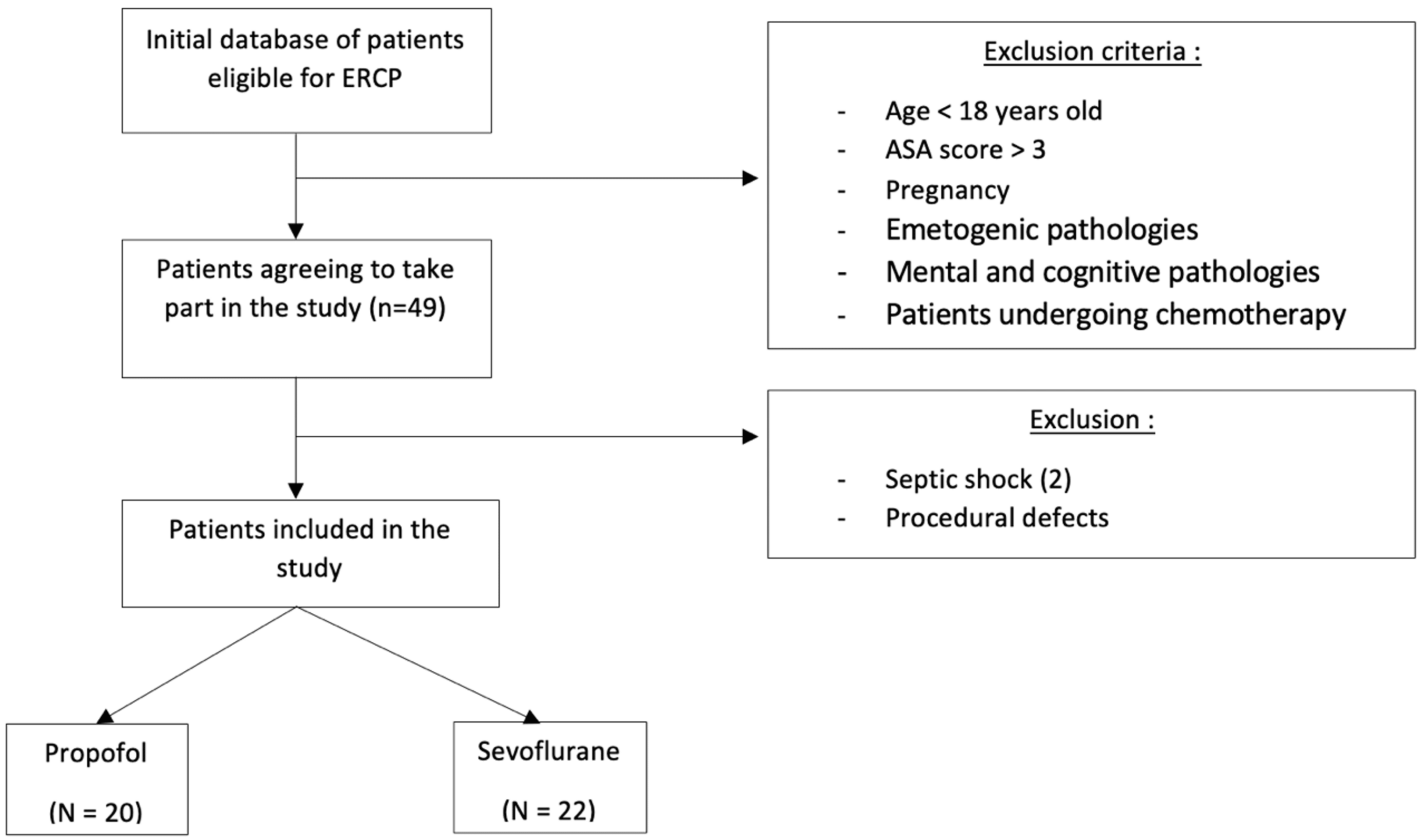
Flowchart showing the composition of the two groups ERCP, endoscopic retrograde cholangio-pancreatography; ASA, American Society of Anesthesiologists

Of the 49 patients considered for the study, seven were excluded from this analysis. Two patients developed septic shock postoperatively requiring intensive care management and five patients had procedural errors (intraoperative injection of a product not authorized by the study). The cohort therefore comprised 42 patients: 22 in the sevoflurane group and 20 in the propofol group (Figure 1).

Study protocol

The patient's informed consent was obtained during the preoperative consultation. Premedication was administered 30 minutes before the procedure with 0.5 mg of sublingual alprazolam. Standard anesthetic monitoring was established (three-lead ECG, pulse oximetry, and non-invasive blood pressure). A peripheral venous line was inserted, and Hartmann’s solution was infused at a rate of 10 mL/kg/h. Pre-oxygenation was performed with a face mask at an FiO₂ of 90% for three minutes.

Anesthesia was induced and maintained according to the previously assigned technique, with an injection of alfentanil 55 µg/kg followed by rocuronium 0.9 mg/kg IV. In the sevoflurane group, induction was achieved with a bolus of propofol at 1.5-2.5 mg/kg IV and maintenance was provided with sevoflurane. In the propofol group, both induction and maintenance were performed using IV propofol administered via TCI (Schnider model) at an effect-site concentration of 3-4 µg/mL.

Mechanical ventilation was adjusted to maintain an end-tidal CO₂ level between 35 and 40 mmHg. During the procedure, mean arterial pressure (MAP) was maintained above 70 mmHg using IV ephedrine or phenylephrine, depending on heart rate.

At the end of the procedure, sugammadex was administered to reverse the effects of rocuronium if necessary. As soon as the patient was extubated, they were transferred to the recovery room with continuous pulse oximetry and oxygen via nasal cannula at 3 L/min. No analgesic or antiemetic prophylaxis was administered preoperatively or intraoperatively.

During the postoperative 48 h, the patient was seen again at 1, 6, 12, 24 and 48 h to ask if they had experienced any nausea or vomiting, for pain assessment using a visual analogue scale (VAS), with the score ranging from 0 (no pain) to 10 (worst pain imaginable), and to collect information on any analgesics and antiemetics received, in accordance with the protocol (see the Appendices). The post-anesthesia care unit (PACU) and ward nurses and doctors were unaware of the anesthesia the patient had received. If the patient was discharged within 48 h, they were contacted to report the analgesics and antiemetics taken at home.

Statistical methodology

All data were collected using REDCap software (Research Electronic Data Capture v14.1.2; Vanderbilt University). The data set was exported to a Microsoft™ Excel™ spreadsheet (Microsoft Corporation, Redmond, USA). Statistical analysis was performed using the NCSS 19.0.3 statistical software (NCSS, LLC, Kaysville, USA). Data are reported as mean ± SD - median (interquartile range), or number of cases N (%), except for data derived from analysis of covariance, which are reported as least-squares mean (±SEM). Numerical data were compared between groups by the non-parametric Mann-Whitney test. Categorical (discrete) data were compared using a chi-square test. The total number of emetic episodes reported at 48 h post-intervention were compared between groups using analysis of covariance with the help of a general linear model, with each patient's Apfel score as a co-variable. A Tukey-Kramer test was used to compare each category of the Apfel score. Numerical pain assessment score values measured at 1, 6, 12, 24 and 48 h post-intervention were compared between groups by repeated measures analysis of variance using a general linear model. For all tests, a value of p<0.05 was considered statistically significant. The variables studied are described in Table 2.

**Table 2:** Summary of variables studied PONV, postoperative nausea and vomiting; GERD, gastroesophageal reflux disease; PUD, peptic ulcer disease; VAS, visual analogue scale; ASA, American Society of Anesthesiologists

Variables	
Demographic and medical variables	Age (years)
	BMI (kg/m²)
	Gender (M/F)
	Oncopathology (yes/no)
	Diabetes (yes/no)
	GERD and/or PUD (yes/no)
Anesthetic and operative variables	ASA score (1/2/3)
	Apfel score (0/1/2/3/4)
	Operating time (min): time from anesthetic injection to patient extubation
Follow-up variables postoperatively	Number of PONV episodes perceived by patients during the 48 h postoperatively
	Number of antiemetics received by patients during the 48 postoperative hours
	Patients’ VAS scores during the five postoperative periods
	Type of analgesic received during the 48 h postoperatively

## Results

Demographic and medical data

Univariate analysis showed no significant difference between the two groups (Table 3).

**Table 3:** Comparison of demographic and medical variables between the two study groups GERD, gastroesophageal reflux disease; PUD, peptic ulcer disease Results are presented as mean ± standard deviation – median (interquartile range). p<0.05 was considered statistically significant.

	Sevoflurane (N=22)	Propofol (N=20)	p
Age (years)	56.5 ± 19.9 – 58.5 (48.5-66)	64.1 ± 16.1 – 65 (56-74.5)	0.12
Body mass index (kg/m²)	25.7 ± 5.7 – 25.2 (21.8-28.5)	25.4 ± 6.2 – 23.6 (21.3-27.0)	0.57
Gender (M/F), n (%)	13 (59.1%)/9 (40.9%)	10 (50%)/10 (50%)	0.55
Cancer (yes/no), n (%)	15 (68.2%)/7 (31.8%)	10 (50%)/10 (50%)	0.23
Diabetes (yes/no), n (%)	5 (23%)/17 (77%)	5 (25%)/15 (75%)	0.86
GERD and/or PUD (yes/no), n (%)	4 (18%)/18 (82%)	4 (20%)/16 (80%)	0.88

Anesthetic and operative data

Univariate analysis showed no significant difference between the two groups (Table 4).

**Table 4:** Comparison of anesthetic and operative variables between the two study groups ASA, American Society of Anesthesiologists Results are presented as mean ± standard deviation – median (interquartile range). p<0.05 was considered statistically significant.

	Sevoflurane (N=22)	Propofol (N=20)	p
Intervention time (min)	70.8 ± 29.1 – 65 (55.5-86.7)	76.9 ± 38.3 – 69.5 (49.7-100)	0.78
ASA score			0.65
1	1 (4.6%)	1 (5%)	-
2	13 (59.1%)	9 (45%)	-
3	8 (36.4%)	10 (50%)	-
Apfel score			0.63
0	7 (31.8%)	5 (25%)	-
1	10 (45.5%)	9 (45%)	-
2	4 (18.2%)	6 (30%)	-
3	1 (4.5%)	0 (0%)	-
4	0 (0%)	0 (0%)	-

Postoperative follow-up variables

Univariate analysis showed no significant difference between the two groups in the occurrence of PONV (Table 5) or in the number of emetic episodes at 48 h postoperatively (Table 6). Analysis of covariance with the Apfel score as a cofactor showed no significant difference between the two anesthesia techniques regarding the occurrence of PONV (Table 7). The analysis showed no significant differences in postoperative pain and no effect of time on pain levels (Table 8). Also, there were no significant differences in the treatments received postoperatively (Table 9).

**Table 5:** Analysis of the effect of anesthesia on the occurrence of postoperative nausea and vomiting (PONV)

	Sevoflurane (N=22)	Propofol (N=20)	p
PONV			0.66
Yes	8 (36.4%)	6 (30%)	-
No	16 (63.6%)	14 (70%)	-

**Table 6:** Analysis of the effect of anesthesia on the number of postoperative nausea and vomiting (PONV) episodes and the consumption of antiemetics Results are presented as mean ± standard deviation – median (interquartile range). p<0.05 was considered statistically significant.

	Sevoflurane (N=22)	Propofol (N=20)	p
No. of emetic episodes at 48 h, whole group	1 ± 1.9 – 0 (0-2)	0.6 ± 1.2 – 0 (0-1)	0.55
No. of emetic episodes at 48 h, patients with PONV	2.75 ± 2.3 – 2 (1.25-3.5)	2 ± 1.5 – 1.5 (1-2.75)	0.41
No. of antiemetics administered, whole group	0.36 ± 0.58 – 0 (0-1)	0.20 ± 0.52 – 0 (0-0)	0.23
No. of antiemetics administered, patients with PONV	1 ± 0.53 – 1 (1-1)	0.67 ± 0.82 – 0.5 (0-1.25)	0.31

**Table 7:** Analysis of the effect of anesthesia on postoperative nausea and vomiting (PONV) episodes during the 48-h postoperative period based on the Apfel score Results are presented as mean ± standard deviation. Effect of anesthesia, p = 0.24; effect of Apfel score, p = 0.51; anesthesia x Apfel score, p = 0.39. p<0.05 was considered statistically significant.

Apfel score	Sevoflurane (N=22)	Propofol (N=20)	p
0	1.06 ± 0.53	0.08 ± 0.63	0.24
1	0.99 ± 0.34	0.57 ± 0.36	0.41
2	0.93 ± 0.56	1.06 ± 0.59	0.87
3	0.86 ± 0.93	1.56 ± 1.03	0.62

**Table 8:** Analysis of the effect of anesthesia on visual analog assessment as a function of time Results are presented as mean ± standard deviation – median (interquartile range). Effect of anesthesia, p = 0.69; time effect, p = 0.051; anesthesia x time, p = 0.66. p<0.05 was considered statistically significant.

	Sevoflurane (N=22)	Propofol (N=20)
Postop 1 h	0.83 ± 1.88 – 0 (0-0.5)	0.95 ± 2.18 – 0 (0-0)
Postop 6 h	1.54 ± 2.04 – 0 (0-3.25)	2.05 ± 2.94 – 0 (0-3)
Postop 12 h	0.36 ± 0.84 – 0 (0-0)	1.05 ± 1.98 – 0 (0-1.75)
Postop 24 h	1.63 ± 2.32 – 0 (0-4)	1.35 ± 1.87 – 0 (0-3)
Postop 48 h	1.09 ± 1.35 – 0 (0-2.25)	0.75 ± 1.77 – 0 (0-0)

**Table 9:** Analysis of the effect of anesthesia on the type of analgesic used postoperatively p<0.05 was considered statistically significant.

	Sevoflurane (N=22)	Propofol (N=20)	p
Type of analgesic used			0.80
No treatment	5 (22.7%)	5 (25%)	-
Paracetamol or tramadol IV or PO	15 (68.2%)	12 (60%)	-
Strong opioid IV or PO	2 (9.1%)	3 (15%)	-

Power calculation

Based on the incidence of postoperative nausea and vomiting observed in the study, the required sample size for a larger study was determined according to the desired type I error rate and statistical power (Table 10).

**Table 10:** Number of patients required according to the type 1 error accepted and the desired test power

Type I error (α)	Test power (1-β)	Number of patients per group
5%	80%	254
5%	90%	341
1%	80%	378
1%	90%	481

## Discussion

The composition of the two groups was homogeneous. There was no significant difference in terms of emetic pathologies accepted in the study. Furthermore, no significant differences were found in ASA scores or duration of surgery, both of which may influence the incidence of PONV [13]. This suggests that the two groups in this cohort are comparable and originate from the same general population.

Regarding PONV, the patients mostly had an Apfel score of 2 or lower, which means the probability of PONV occurrence is between 20% and 40% according to the scientific literature [2-5,14]. In the results presented above, the incidence of PONV was similar across the entire cohort as well as within each of the two separate groups. This demonstrates that PONV remains a frequent problem, even in the specific setting of interventional endoscopy, even though ERCPs were performed by skilled operators to limit intervention time. However, it is possible that intra-duodenal CO_2_ insufflation may have played a role in the incidence of PONV [15,16].

On the other hand, this study did not demonstrate a significant difference between TIVA and inhaled anesthesia in terms of PONV incidence or the number of emetic episodes at 48 h postoperatively. Additionally, the same result was found for the use of postoperative antiemetics. These results contradict what is described in the scientific literature, which indicates that the use of volatile anesthetics is associated with an increased incidence of PONV [17] and propofol reduces it through an effect on dopaminergic receptors [18]. However, these studies were conducted on surgical interventions, raising doubt about the applicability of this difference to interventional endoscopy. Moreover, the analysis of covariance showed no significant effect of the Apfel score on the occurrence of PONV, which is surprising because patient-related risk factors should be represented in the sample if it reflects the general population. Therefore, we would expect to find that the higher the Apfel score, the higher the incidence of PONV. As a result, we can conclude that the statistical analysis may lack power due to the small number of patients studied. This preliminary study allows to highlight a slightly lower trend in the TIVA group, but without a significant difference.

Regarding POP, the analysis of the VAS during the 48 h postoperatively showed no significant difference between the two groups. The trend indicated generally mild pain with a non-significant time effect. Furthermore, there was no significant difference in the use of analgesics postoperatively. These results show that the groups were treated similarly for pain and that there was no significant over- or underuse of opioids, which could generate PONV in one group and not the other. This result is interesting because it is found that halogenated agents may have an anti-nociceptive effect with or without second gas effect [19,20]. Nevertheless, in clinical practice, this difference is not found [21]. It should be noted that the studies were conducted on open or laparoscopic surgeries, but never on endoscopic procedures, which are generally shorter and less invasive.

Limitations

This study was not without limitations. First, this was a monocentric study with a small sample size. Moreover, the Apfel score, which plays a major role in the incidence of PONV [22], was relatively low in the population studied, limiting the appearance of differences between the two groups. Hence, a study with a larger sample size is required to confirm the results. Sample size calculation for a larger follow-up study was carried out according to the alpha level and the desired power of the study (Table 10).

## Conclusions

This study therefore clearly shows that, at this stage, there are no differences between inhaled anesthesia and TIVA for interventional gastroenterology procedures, with regard to PONV and POP. However, the results of this study are preliminary and still need to be confirmed by subsequent studies with much larger sample sizes.

## Appendices

**Table 11:** Emetogenic pathologies

System	Acute	Chronic
Digestive	Gastroenteritis, food poisoning, intestinal obstruction, biliary colic, appendicitis, peritonitis, cholecystitis, hepatitis, acute pancreatitis, abdominal radiotherapy	Gastroparesis, gastroesophageal reflux disease, gastric ulcer, pyloroduodenal stenosis, eosinophilic gastroenteritis
Uro-genital	Renal colic, adnexal torsion, ectopic pregnancy	
Cardiovascular	Myocardial infarction, aortic dissection, intestinal ischemia	Chronic heart failure
ENT	Vestibular neuritis, Meniere's disease	
Neurological	Intracranial hypertension, subarachnoid hemorrhage, meningitis, cerebellar disease, migraine, cerebral radiotherapy	
Metabolic	Acute renal failure, acute adrenal insufficiency, acute hyperthyroidism	Diabetes, diabetic ketoacidosis in pregnancy, chronic renal failure, uricemia, hypercalcemia, hyponatremia, alcoholic disease
Medicinal	Erythromycin, aminosides, colchicine, levodopa	Levodopa drug intoxication
Psychiatric		Anorexia nervosa, bulimia nervosa, depression, psychogenic vomiting
Other	Systemic infection, acute glaucoma	Chronic nausea and vomiting syndrome

**Table 12:** Chemotherapies excluded and the degrees of emetogenicity

Degrees of emetogenicity	Agents
High (>90%)	Cisplatin, cyclophosphamide >1500 mg/m², dacarbazine, doxorubicin/epirubicine + cyclophosphamide
Moderate (30%-90%)	Oxaliplatin, carboplatin, cyclophosphamide <1500 mg/m², doxorubicin, epirubicin, irinotecan
Low (10%-30%)	Docetaxel, paclitaxel, doxorubicin, liposomal etoposide, pemetrexed, methotrexate, 5-fluorouracil gemcitabine
Minimal (<1%)	Vinorelbine, bleomycin

**Table 13:** Apfel score

Risk factors	Points
Woman	1
Non-smoking	1
Antecedents of nausea and vomiting or motion sickness	1
Postoperative opioid planned	1

**Table 14:** ASA score ASA, American Society of Anesthesiologists

ASA score	Description
1	Normal patient
2	Patients with moderate systemic abnormalities
3	Patients with severe systemic abnormalities
4	Patients with severe, life-threatening systemic abnormalities
5	Moribund patient unlikely to survive without intervention
6	Brain-dead patient whose organs are removed for transplantation

**Table 15:** PONV management protocol according to the Apfel score PONV, postoperative nausea and vomiting

Score 0 to 2	Score = 3	Score = 4
Alizapride 50 mg or dehydrobenzperidol 1.25 mg, or ondansetron 4 mg	Alizapride 100 mg + ondansetron 4 mg	Alizapride 100 mg + haloperidol 1 mg

## References

[1] Robinson DH, Toledo AH (2012). Historical development of modern anesthesia. J Invest Surg.

[2] Macario A, Weinger M, Carney S, Kim A (1999). Which clinical anesthesia outcomes are important to avoid? The perspective of patients. Anesth Analg.

[3] Lerman J (1992). Surgical and patient factors involved in postoperative nausea and vomiting. Br J Anaesth.

[4] Habib AS, Gan TJ (2004). Evidence-based management of postoperative nausea and vomiting: a review. Can J Anaesth.

[5] Horn CC, Wallisch WJ, Homanics GE, Williams JP (2014). Pathophysiological and neurochemical mechanisms of postoperative nausea and vomiting. Eur J Pharmacol.

[6] Cao X, White PF, Ma H (2017). An update on the management of postoperative nausea and vomiting. J Anesth.

[7] Watcha MF, White PF (1992). Postoperative nausea and vomiting. Its etiology, treatment, and prevention. Anesthesiology.

[8] Sneyd JR, Carr A, Byrom WD, Bilski AJ (1998). A meta-analysis of nausea and vomiting following maintenance of anaesthesia with propofol or inhalational agents. Eur J Anaesthesiol.

[9] Kim GH, Ahn HJ, Kim HS, Bang SR, Cho HS, Yang M, Kim JA (2011). Postoperative nausea and vomiting after endoscopic thyroidectomy: total intravenous vs. balanced anesthesia. Korean J Anesthesiol.

[10] Mukherjee K, Seavell C, Rawlings E, Weiss A (2003). A comparison of total intravenous with balanced anaesthesia for middle ear surgery: effects on postoperative nausea and vomiting, pain, and conditions of surgery. Anaesthesia.

[11] Masci E, Toti G, Mariani A (2001). Complications of diagnostic and therapeutic ERCP: a prospective multicenter study. Am J Gastroenterol.

[12] Vandervoort J, Soetikno RM, Tham TC (2002). Risk factors for complications after performance of ERCP. Gastrointest Endosc.

[13] Gan TJ (2006). Risk factors for postoperative nausea and vomiting. Anesth Analg.

[14] Eberhart LH, Morin AM (2011). Risk scores for predicting postoperative nausea and vomiting are clinically useful tools and should be used in every patient: con - 'life is really simple, but we insist on making it complicated'. Eur J Anaesthesiol.

[15] Shinn HK, Lee MH, Moon SY, Hwang SI, Lee CS, Lim HK, Song JH (2011). Post-operative nausea and vomiting after gynecologic laparoscopic surgery: comparison between propofol and sevoflurane. Korean J Anesthesiol.

[16] Koivusalo AM, Kellokumpu I, Lindgren L (1997). Postoperative drowsiness and emetic sequelae correlate to total amount of carbon dioxide used during laparoscopic cholecystectomy. Surg Endosc.

[17] Boccara G, Mann C, Pouzeratte Y, Bellavoir A, Rouvier A, Colson P (1998). Improved postoperative analgesia with isoflurane than with propofol anaesthesia. Can J Anaesth.

[18] Appadu BL, Strange PG, Lambert DG (1994). Does propofol interact with D2 dopamine receptors?. Anesth Analg.

[19] Ganjoo P, Farber NE, Schwabe D, Kampine JP, Schmeling WT (1996). Desflurane attenuates the somatosympathetic reflex in rats. Anesth Analg.

[20] O'Connor TC, Abram SE (1995). Inhibition of nociception-induced spinal sensitization by anesthetic agents. Anesthesiology.

[21] Fassoulaki A, Melemeni A, Paraskeva A, Siafaka I, Sarantopoulos C (2008). Postoperative pain and analgesic requirements after anesthesia with sevoflurane, desflurane or propofol. Anesth Analg.

[22] Apfel CC, Heidrich FM, Jukar-Rao S (2012). Evidence-based analysis of risk factors for postoperative nausea and vomiting. Br J Anaesth.

